# Knowledge of infection prevention and control among healthcare workers and factors influencing compliance: a systematic review

**DOI:** 10.1186/s13756-021-00957-0

**Published:** 2021-06-03

**Authors:** Saad Alhumaid, Abbas Al Mutair, Zainab Al Alawi, Murtadha Alsuliman, Gasmelseed Y. Ahmed, Ali A. Rabaan, Jaffar A. Al-Tawfiq, Awad Al-Omari

**Affiliations:** 1 Administration of Pharmaceutical Care, Al-Ahsa Health Cluster, Ministry of Health, Al-Ahsa, Saudi Arabia; 2 Research Center, Almoosa Specialist Hospital, Dhahran Street, Al-Ahsa, 31982 Saudi Arabia; 3 College of Nursing, Princess Nourah Bint Abdul Rahman University, Riyadh, Saudi Arabia; 4 School of Nursing, University of Wollongong, Wollongong, Australia; 5 Department of Paediatrics, College of Medicine, King Faisal University, Al-Ahsa, Saudi Arabia; 6 Department of Pharmacy, Hereditary Blood Diseases Centre, Al-Ahsa, Saudi Arabia; 7 Molecular Diagnostic Laboratory, Johns Hopkins Aramco Healthcare, Dhahran, Saudi Arabia; 8 Infectious Disease Unit, Specialty Internal Medicine, Johns Hopkins Aramco Healthcare, Dhahran, Saudi Arabia; 9 Infectious Disease Division, Department of Medicine, Indiana University School of Medicine, Indianapolis, IN USA; 10 Infectious Disease Division, Department of Medicine, Johns Hopkins University School of Medicine, Baltimore, MD USA; 11 College of Medicine, Alfaisal University, Riyadh, Saudi Arabia; 12 Research Center, Dr. Sulaiman Al Habib Medical Group, Riyadh, Saudi Arabia

**Keywords:** Awareness, Adherence, Compliance, Control, Factors, Healthcare, Infection, Knowledge, Prevention, Workers

## Abstract

**Background:**

Knowledge of infection prevention and control (IPC) procedures among healthcare workers (HCWs) is crucial for effective IPC. Compliance with IPC measures has critical implications for HCWs safety, patient protection and the care environment.

**Aims:**

To discuss the body of available literature regarding HCWs' knowledge of IPC and highlight potential factors that may influence compliance to IPC precautions.

**Design:**

A systematic review. A protocol was developed based on the Preferred Reporting Items for Systematic reviews and Meta-Analysis [PRISMA] statement.

**Data sources:**

Electronic databases (PubMed, CINAHL, Embase, Proquest, Wiley online library, Medline, and Nature) were searched from 1 January 2006 to 31 January 2021 in the English language using the following keywords alone or in combination: *knowledge, awareness, healthcare workers, infection, compliance, comply, control, prevention, factors*. 3417 papers were identified and 30 papers were included in the review.

**Results:**

Overall, the level of HCW knowledge of IPC appears to be adequate, good, and/or high concerning standard precautions, hand hygiene, and care pertaining to urinary catheters. Acceptable levels of knowledge were also detected in regards to IPC measures for specific diseases including TB, MRSA, MERS-CoV, COVID-19 and Ebola. However, gaps were identified in several HCWs' knowledge concerning occupational vaccinations, the modes of transmission of infectious diseases, and the risk of infection from needle stick and sharps injuries. Several factors for noncompliance surrounding IPC guidelines are discussed, as are recommendations for improving adherence to those guidelines.

**Conclusion:**

Embracing a multifaceted approach towards improving IPC-intervention strategies is highly suggested. The goal being to improve compliance among HCWs with IPC measures is necessary.

## Introduction

Healthcare-associated infections (HAIs) are a major problem for patients' and healthcare workers' (HCWs') safety and their prevention must be a top priority for healthcare systems and organizations [1–4]. HAIs prevalence ranges from 5 to 15% of hospitalized patients and can affect 9–37% of those admitted to intensive care units (ICUs) [5]. At any one time in the United States (US), 1 out of every 25 hospitalized patients are affected by a HAI [6].

**Fig. 1:**
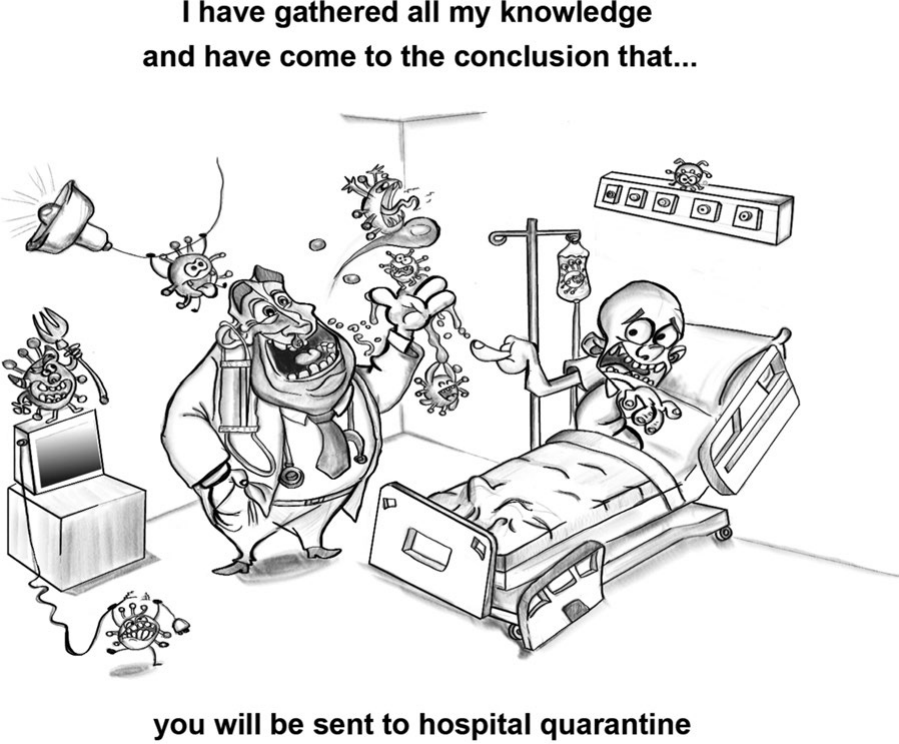
A caricature depicts patient’s intense feeling of fear about a HCW being not compliant with IPC during the consultation process

HAIs can result in low quality of life, or even reduce life expectancy of the infected person, as well as incur considerable costs in the long run [4, 7–9]. For example, the risk of HAIs following a needle-stick injury with needle from an infected source patient was 0.3% for HIV, 3% for hepatitis C and 6–30% for hepatitis B [10]. A total of 3 million out of 35 million HCWs worldwide experienced percutaneous exposure to bloodborne pathogens (BBPs) each year; 2 million of those were to HBV; 0.9 million to HCV; and 0.17 million to HIV [11]. The annual economic impact of HAIs in the US alone was approximately US$ 6.5 billion [9]. HAIs have also been reported to contribute to serious mental health disorders, including anxiety, depression, adjustment disorder, panic attacks, and post-traumatic stress disorder [12, 13]. Figure 1 illustrates patient's worriment about HCW's noncompliance with the IPC. The size and scope of the HAIs burden worldwide appears to be very important and quite underestimated. Methods to assess the size and nature of the problem exist, however, these tools need to be simplified and adapted so as to be affordable in settings where resources and data sources are limited. Similarly, preventive measures are often simple to implement, such as hand hygiene. IPC must reach a higher position among the first priorities in national health programmes, especially in resource constrained countries [5].

Luckily, as many as 55–70% of HAIs may be preventable [4]. To prevent HAIs, measures such as standard precautions (including hand hygiene, use of gloves, gowns, eye protection, use of cough etiquette, and safe disposal of sharp instruments) and isolation precautions used to interrupt the risk of transmission of pathogens (contact, droplet, and airborne precautions) are recommended and implemented widely [14]. Prevention of specific infections, prophylaxis after exposure to BBPs and immunizations for HCWs are another IPC measures followed to reduce the rate of HAIs [14].

Knowledge of HCWs is fundamental for effective IPC [5, 11, 15]. Lack of knowledge of guidelines for IPC—combined with an unawareness of preventive indications during daily patient care and the potential risks of transmission of microorganisms to patients—constitute barriers to IPC compliance [16–18]. Lack of knowledge about the appropriateness, efficacy and use of IPC measures determine poor compliance [19–22]. To overcome these barriers, education and training are the cornerstones of improvement in IPC practices [23, 24]. HCWs should be aware of the fact that knowledge is power. However, lack of knowledge of IPC measures has been repeatedly shown after education and training [24, 25]. HCWs' awareness should include issues related to hand hygiene, wearing personal protective equipment (PPE), immunization for prevention of communicable diseases, modes of infection transmission, assessment of patients for infection, medical instrument decontamination, healthcare waste handling, and needle stick and sharp safety policy. Even more importantly, HCWs should be compliant to these IPC precautions, methods and strategies to ensure HAIs reduction in the healthcare settings [20].

Compliance with IPC practices, including hand hygiene and use of PPE, has been found to vary widely among HCWs [20, 26, 27] and is likely influenced by one’s knowledge about infection risk and behaviours [16, 18, 27–32]. However, good knowledge does not necessarily predict good IPC practice [8, 33, 34]. For example, HCWs have been found to demonstrate poor compliance with hand hygiene practices despite well-established guidelines for the prevention of HAIs [35, 36].

More confounding variables of good IPC practice other than knowledge or experience exist.

Given the potential negative impacts on patients and HCWs by HAIs, clinical and national economic and psychological burden as mentioned, it is important to discuss literature on HCWs' knowledge of IPC to prevent such harmful exposures. Moreover, this paper will also focus on reviewing potential factors influencing compliance of HCWs with the IPC measures so that some suggestions can be made to improve the quality and safety of health service delivery and the health outcomes of the people who access those services.

## Methods

### Design

This systematic review was conducted with reference to the basics of Cochrane Handbook for Systematic Reviews of Interventions [37], described as stated by the Preferred Reporting Items for Systematic reviews and Meta-Analysis [PRISMA] statement [38]. A systematic review protocol was developed based on PRISMA-P and the PRISMA statement. Published articles in English from 1 January 2006, to 31 January 2021, were retrieved for review from 7 electronic databases (PubMed, CINAHL, Embase, Proquest, Wiley online library, Medline, and Nature). Search terms included *knowledge*, *awareness*, *healthcare workers*, *infection*, *control*, *comply*, *compliance*, *prevention* and *factors*. Relevant papers were identified by three independent readers using predefined exclusion criteria, firstly on the basis of abstracts, secondly by assessing full-text papers. The title and abstract of each retrieved article were read, and the article was retained if it discussed HCW's knowledge of IPC or highlighted likely factors influencing compliance to the IPC precautions.

### Inclusion–exclusion criteria

Articles were eligible for inclusion in this review when they met all of these criteria: (1) reported on HCWs' knowledge and/or compliance of IPC; (2) used a quantitative, qualitative or combined method; and (3) published between January 2006 and January 2021 in English. Articles were excluded if they met one of the following criteria: (1) editorials, commentaries, news analyses, reviews and systematic reviews or meta-analyses; (2) small sample size (studies with respondents of ≤ 100); or (3) undertaken repeated methods (similar outcome measures, design, survey questionnaire tools and/or respondents of the study). Studies involving the following group of HCWs were included in the review: physicians, nurses, nurse assistants, pharmacists and pharmacy technicians, midwives, laboratory specialists and technicians, laboratory technologists, radiographers, community health workers, health officers, hospital orderlies, and other healthcare professionals.

Topics of interest for the outcomes’ measures were: standard or universal precautions (hand hygiene; wearing PPE, glove use, mask use, and protective eyewear use; sharps safety; safe injection practices; and sterile instruments and devices). Comparable outcomes on respiratory hygiene IPC measures designed to limit the transmission of respiratory pathogens spread by droplet or airborne routes [tuberculosis (TB), methicillin-resistant staphylococcus aureus (MRSA), Middle East Respiratory Syndrome (MERS-CoV), coronavirus disease 2019 (COVID-19) and Ebola] were included. Findings on HCWs knowledge of IPC measures necessary to stop HAIs like occupational vaccinations (HBV, varicella, influenza and COVID-19); and infections transmitted through needle stick and sharp injuries (NSSIs), awareness of national injection safety policy, and healthcare waste handling, care to prevent urinary catheters- and central venous catheters (CVCs)-related infections were also considered. Both observed and self-report measures of these outcomes were examined.

### Data extraction

Three researchers (S.A., A.A. and A.R.) independently screened titles and abstracts of the retrieved studies for eligibility. The full text was then reviewed to confirm an eligibility criteria match. Disagreements between the three reviewers after full text screening were reconciled via consensus by fourth, fifth and sixth reviewers (Z.A., G.A. and J.A.). Data were extracted from the relevant research studies using key headings which are noted in Tables 1 and 2, simplifying analysis and review of the literature. Articles were categorized as a survey report, an observational study, or a semi-structured interview study. The following data were extracted from retrieved studies: authors, publication year, study location and aim, setting, sample size, methodology and assessment of study risk of bias, and outcome. Appropriate quality appraisal guides and checklists were used to evaluate the quality of the survey and observational studies and the semi-structured interviews studies [39, 40] involved in this review. Three investigators (S.A., A.A., and Z.A.) separately evaluated the possibility of bias using these tools. Quality assessment items were based on research problems, research design, study sample, data collection, results, and limitations.

**Table 1:** Summary of the characteristics of the included studies that have assessed the knowledge of IPC among HCWs (n = 25), 2006–2021

Author, year, study location	Study aim	Setting	Responded population	Methodology; and [assessment of study risk of bias (tool used; finding)]	Key findings
Abeje et al. [28], Ethiopia	Evaluate hepatitis B vaccination knowledge among HCWs	Multi-centre	374 HCWs (nurses, health officers, medical doctors, dentists, and laboratory technologists)	Survey: cross-sectional questionnaire; [(Williamson critical appraisal of qualitative evidence, LOW risk of bias)]	HCWs who scored above the mean (mean knowledge score of respondents was 7.6) were classified knowledgeable using a questionnaire tool with a total score ranged from 0 to 10. Only 52% of the respondents were knowledgeable about hepatitis B infection and 62% of HCWs were knowledgeable about hepatitis B vaccine
Albano et al. [16], Italy	Assess knowledge towards influenza A/H1N1 and the vaccination among HCWs	Multi-centre	600 HCWs (physicians, nurses and others)	Survey: cross-sectional questionnaire; [(Hoy critical appraisal checklist, LOW risk of bias)]	Only 36.1% knew the main modes of transmission, and that HCWs are a risk category. Level of knowledge was significantly higher in HCWs having received information through scientific journals (OR = 1.63; 95% CI 1.12–2.38) Majority acquired knowledge from public-media (63.5%), followed by health-care professionals (47.1%), and the internet (45%)
Alsahafi et al. [43], Saudi Arabia	Assess knowledge of HCWs to MERS coronavirus	Multi-centre	1216 (687 nurses, 267 physicians, and 262 other HCWs)	Survey: questionnaire; [(Hoy critical appraisal checklist, LOW risk of bias)]	Majority of HCWs correctly identified patient risk factors (i.e., 88% of the physicians, 69.5% of the nurses and 62.5% of the other HCWs; *p* < 0.001). A low percentage of HCWs was aware that asymptomatic MERS-CoV was described (i.e., 47.6% of the physicians, 30.4% of the nurses and 29.9% of the other HCWs; *p* < 0.001) The most common sources of MERS-CoV information were the Ministry of Health (MOH) memo (74.3%) and MOH web page (72.4%), with smaller proportions reporting use of the MOH Helpline (43.8%) and medical journals (48.2%) Majority of the HCWs (≥ 72.3%) indicated that that they are in need for educational courses and training about the MERS-CoV, Ebola and other emerging infectious diseases Only 22.8% reported having received training about dealing with infectious disease outbreaks, 37.1% reported training in infection control policies and procedures, 54.4% reported training in hand hygiene and 45.6% reported training in N95 mask wearing techniques
Amoran et al. [53], Nigeria	Assesses level of knowledge with standard precautions by HCWs	Single centre	421 HCWs (52 doctors, 78 nurses, 54 laboratory scientists, 53 pharmacists, 57 community health workers, 74 hospital orderlies, and 53 other professions)	Survey: cross-sectional questionnaire; [(Williamson critical appraisal of qualitative evidence, LOW risk of bias)]	Majority (77.9%) of HCWs were able to correctly describe universal precaution and IPC. Some of the HCWs could not recognize vaccination (19.2%), PEP (19.2%), and surveillance for emerging diseases (28.0%) as standard precaution for IPC. Only 2.1% of HCWs were aware of National Injection Safety Policy and 1.9% were aware of Policy on Sharps Disposal Only 8.1% of HCWs had attended any workshop or training in IPC in the last 2 years and almost all of the HCWs admitted training needs on IPC
Arora et al. [29], India	Assess knowledge of HCWs about the NSSIs	Single centre	190 HCWs (50 doctors, 100 nurses, 15 technicians, and 25 housekeeping staff)	Survey: cross-sectional questionnaire; [(Hoy critical appraisal checklist, LOW risk of bias)]	Majority (94.7%) were aware about standard precautions. Only 50.2% HCWs gave correct answers regarding disease transmission through NSSIs
Ashraf et al. [30], United States	Assess knowledge of 2002 CDC hand hygiene guidelines	Multi-centre	1143 HCWs (386 nursing assistants, 375 nurses, and 382 other healthcare professionals)	Survey: questionnaire; [(Hoy critical appraisal checklist, MODERATE risk of bias)]	Most HCWs (83.6%) reported familiarity with the CDC guidelines. Nurses were more likely to answer most of the questions correctly, compared with nursing assistants and other professionals (*p* values < 0.05) About (20.8%) of HCWs did not receive any hand hygiene training or orientation in the prior year or were not sure whether they had received training
Assefa et al. [17], Ethiopia	Evaluate knowledge of HCWs about hand hygiene practices, utilization of PPE, and PEP, healthcare waste management practices, and instrument disinfection practice	Multi-centre	171 HCWs (about 83 were nurses)	Survey: questionnaire; [(Williamson critical appraisal of qualitative evidence, LOW risk of bias)]	About 70.8% of HCWs had adequate knowledge (i.e., a HCW score on IPC knowledge was equal or above the mean) About 19.3% of HCWs didn’t take any training on IPC and universal precautions Having IPC guideline (AOR = 3.65, 95% CI 1.26, 10.54), taking IPC training (AOR = 2.2, 95% CI 1.01, 4.75), having 5 years or more work experience (AOR = 1.52: 95% CI 1.13, 4.51), and working in maternity unit (AOR = 1.67; 95% CI 1.38–5.23) were positively associated with adequate knowledge of IPC
Chuc et al. [31], Vietnam	Assess and compare HCWs knowledge and self-reported practices of IPC in a rural and an urban hospital	Multi-centre	339 HCWs (nurses, midwives, physicians and cleaners)	Survey: cross-sectional questionnaire; [(Hoy critical appraisal checklist, LOW risk of bias)]	Majority of HCWs had good (i.e., a total score of 7.5 to < 11.25) or adequate (i.e., a total score ≥ 11.25) knowledge using a questionnaire tool with a total score ranged from 0 to 15 Cleaners had lower knowledge than both physicians and nurses [OR (95% CI ): 0.13 (0.04–0.51), *p* = 0.001 and 0.12 (0.03–0.41), *p* < 0.001] compared to physicians and nurses/midwives, respectively
Desta et al. [54], Ethiopia	Examine the knowledge and practice of HCWs on IPC and its associated factors among health professionals	Single centre	150 HCWs (21 Physician, 83 nurses, 18 midwives, 3 health officers, 13 lab technicians, 12 others)	Survey: cross-sectional questionnaire; [(Hoy critical appraisal checklist, LOW risk of bias)]	Majority (84.7%) of the HCWs in the hospitals had adequate knowledge on IPC HCWs with experience of above 10 years was four times more likely knowledgeable on IPC than those had work experience of fewer than 5 years (AOR = 4.03, 95% CI = [1.229–5.68]) HCWs with an educational level of master or above and were three times (AOR = 3.034, 95% CI = [1.856–4.756]) and bachelor were two times (AOR = 2.15, 95% CI = [3.245–8.789]) more likely knowledgeable than diplomas Furthermore, HCWs who haven’t taken IPC training were 75% less likely knowledgeable (AOR = 0.25, 95% CI = [1.689–3.95]) about IPC than those had taken training in IPC
Douville et al. [52], United States	Determine knowledge of children's hospital HCWs toward mandatory influenza vaccination	Single centre	585 HCWs (physicians, nurses, and all other hospital employees)	Survey: questionnaire; [(Hoy critical appraisal checklist, LOW risk of bias)]	Comparing those who favored a mandate with those who opposed one, knowledge about CDC recommendations was high for both groups (i.e., difference on knowledge of two variables: 89.3% vs 90.1%, *p* = 0.82; and 83.6% vs 80.2%, *p* = 0.46; respectively) and there were no significant differences in knowledge about the dangers of influenza for patients (i.e., difference on knowledge of two variables: 59.2% vs 61.3%, *p* = 0.8; and 70.5% vs 61.7%, *p* = 0.12; respectively)
Geberemariyam et al. [18], Ethiopia	Assess knowledge of HCWs towards IPC	Multi-centre	648 HCWs (physicians, nurses, midwives, anesthetists, laboratory technicians, laboratory technologists, pharmacists, pharmacy technicians, and radiographers)	Survey: cross-sectional questionnaire; [(Hoy critical appraisal checklist, LOW risk of bias)]	HCWs who scored above the mean were classified knowledgeable using a questionnaire tool with a total score ranged from 0 to 10 Only 53.7% (95% CI 49.8–57.4) of the HCWs were assessed as knowledgeable (if score was above the mean). HCWs were more likely to have IPC knowledge if they worked longer 10 years or more (AOR = 3.41; 95% CI 1.22–9.55), worked in facilities with IPC committees (AOR = 1.78; 95% CI 1.01–3.13), had IPC guidelines available (AOR = 3.34; 95% CI 1.65–6.76) and had training (AOR = 5.02, 95% CI :1.45–8.59)
Iliyasu et al. [8], Nigeria	Explore the knowledge of IPC among HCWs in a tertiary referral center	Single centre	200 HCWs (152 nurses and 48 doctors)	Survey: cross-sectional questionnaire; [(Hoy critical appraisal checklist, LOW risk of bias)]	Most HCWs (87.9%) correctly identified hand hygiene as the most effective method to prevent HAIs, with nurses having better knowledge (91%, *p* = 0.001). Only (44.4%), (61.6%), and (42.4%) of the HCWs were aware of the risks of infection following exposure to HIV, HBV and HCV-infected blood, respectively
Labeau et al. [41], 22 European countries	Determine European ICU nurses’ knowledge of guidelines for preventing CVCs-related infection from the CDC	Multi-centre	3405 European ICU nurses	Multi-country survey (October 2006–March 2007). Using a multiple-choice test, knowledge of the ten recommendations for CVCs-related IPC was evaluated; [(Hoy critical appraisal checklist, MODERATE risk of bias)]	The mean score was 4.44 on ten questions. Only 56% knew that CVCs should be replaced on indication only. About 26% recognized that both polyurethane and gauze dressings are recommended. Only 14% checked 2% aqueous chlorhexidine as the recommended disinfection solution. Only 26% knew sets should be replaced every 96 h when administering neither lipid emulsions nor blood products. Experienced nurses performed significantly better than less experienced nurses (*p* < 0.001 for < 5 years vs. > 5 years ICU experience). Nurses from larger ICUs scored significantly lower than nurses from smaller units (*p* < 0.001 for < 8 vs. > 8 beds and for < 15 vs. > 15 beds, respectively)
Loulergue et al. [51], France	Evaluate HCWs knowledge regarding occupational vaccinations (HBV, varicella and influenza)	Single centre	580 HCWs (physicians, nurses, nurses’ assistants)	Survey: cross-sectional questionnaire; [(Hoy critical appraisal checklist, LOW risk of bias)]	Knowledge about the occupational vaccinations of HCWs was low (i.e., 25% of the HCWs were able to list correctly the three mandatory vaccines). Pediatric staff was more aware of influenza and pertussis immunizations (*p* < 0.05). Physicians and nurses have better knowledge about influenza vaccine recommendations than the others (60.4% and 32.7%, respectively, *p* < 0.05). About 45% of HCWs could not cite any recommended vaccinations and 32% cited a mandatory vaccine as recommended Influenza vaccination was associated with knowledge of vaccine recommendations [OR = 1.75, 95% CI 1.13–2.57] and contact with patients [OR = 3.05, 95% CI 1.50–5.91]
Michel-Kabamba et al. [42], Democratic Republic of the Congo	HCWs knowledge on COVID-19-related clinical manifestations and patient care approach was assessed using WHO’s “Exposure Risk Assessment in the Context of COVID-19” questionnaire	Multi-centre	613 HCWs (27.2% were medical doctors and 72.8% were other categories of HCWs)	Survey: cross-sectional questionnaire; [(Hoy critical appraisal checklist, MODERATE risk of bias)]	Over 80% of HCWs had sufficient knowledge on: COVID19 symptoms (89.2% of doctors vs. 80.7% of other HCWs; *p* < 0.05) and patient care approach (89.8% of doctors vs. 83.8% of other HCWs; *p* < 0.05) Only 41.9% of HCWs had attended a lecture, meeting, or discussion about COVID-19 Most of the HCWs mostly used the news media and social media as primary sources of information on COVID-19, whereas the government’s and WHO’s websites were used less COVID-19 knowledge was positively associated with the COVID-19-related IPC practices (AOR: 3.45 ± 2.40; 95% CI 1.88–13.49; *p* < 0.05)
Mody et al. [27], United States	Assess knowledge of recommended urinary catheter care practices among nursing home HCWs	Multi-centre	356 HCWs (127 nurses and 229 nurse aides)	Survey: questionnaire; [(Hoy critical appraisal checklist, LOW risk of bias)]	More than 90% of HCWs were aware of measures such as cleaning around the catheter daily, glove use, and hand hygiene with catheter manipulation. HCWs were less aware of research‐proven recommendations of not disconnecting the catheter from its bag (59% nurses vs. 30% aides, *p* < 0.001), not routinely irrigating the catheter (48% nurses vs. 8% aides, *p* < 0.001), and hand hygiene after casual contact (60% nurses vs. 69% aides, *p* = 0.07). HCWs were also unaware of recommendations regarding alcohol-based hand rub (27% nurses and 32% aides with correct responses, *p* = 0.38) With respect to urinary catheter care, about 52% and 24% of HCWs reported that they learned from didactic formal [in-services, lectures, and nursing school and nurse aides' courses] and informal [prior experience, nurse supervisors, co-workers, and facility policies] methods, respectively; and 24% gained their knowledge both informally and formally Regarding hand hygiene, 51% reported that they learned from didactic formal methods, 15% for informal methods, and 34% gained their knowledge by both informal and formal methods
Ogoina et al. [34], Nigeria	Examine knowledge of some components of standard precautions among HCWs in two tertiary hospitals	Multi-centre	290 HCWs (111 doctors, 147 nurses and 32 laboratory scientists)	Survey: cross-sectional questionnaire; [(Hoy critical appraisal checklist, LOW risk of bias)]	Overall median knowledge scores toward standard precautions were above 90%. Majority of the HCWs had poor knowledge of injection safety (50% of participants were ignorant of the WHO’s recommendation that sharps/needles should never be recapped, bent or broken) Knowledge of medical laboratory scientists was significantly lower than that of the principal nursing officer/chief nursing officer (85% vs 95%, *p* = 0.027) and the knowledge of the staff nurse/senior nursing officer (90%) were also significantly lower than those of the principal nursing officer/chief nursing officer (*p* = 0.049) About 51.4% of HCWs never had training on IPC and HCWs who had prior IPC training had significantly higher median knowledge percentage scores than those who did not have prior training [median: 95% vs 90%, IQR: 75–95; *p* = 0.002]
Parmeggiani et al. [3], Italy	Assess HCWs knowledge on IPC in the EDs	Multi-centre	307 HCWs (nurses, physicians and other healthcare professionals)	Survey: cross-sectional questionnaire; [(Hoy critical appraisal checklist, LOW risk of bias)]	Majority (87.9%) were aware that HCWs can acquire HCV and HIV from a patient, but less than one-third knew that HCWs can transmit these infections to a patient. Majority identified as proper HAIs control measures the use of gloves, mask, and protective eye wear (94.1%) and hands hygiene measures after removing gloves (91.5%). Overall, 86.3% were aware of both preventive measures and this knowledge was significantly higher in nurses (OR = 2.34, 95% CI 1.09–5.01, *p* = 0.029) Knowledge of proper HAIs IPC measures was significantly higher in HCWs who received information about HAIs from educational courses and scientific journals (OR = 3.54; 95% CI 1.47–8.5). Furthermore, HCWs who have received information about HAIs from educational courses and scientific journals (OR = 3.54; 95% CI 1.22–10.24), and who did not need additional information about HAIs (OR = 0.06; 95% CI 0.01–0.55) were more likely to know the risk for a HCW of acquiring both HCV and HIV from a patient Sources of information about HAIs were: educational courses (71%), and scientific journals (48.2%); however, 85.3% of HCWs claimed to need to update what they already knew
Paudyal et al. [44], Nepal	Assess HCWs knowledge on IPC in the acute care hospitals	Multi-centre	324 HCWs (158 doctors and 166 nurses)	Survey: questionnaire; [(Hoy critical appraisal checklist, LOW risk of bias)]	Although mean knowledge scores were high, only 16% answered the entire knowledge section correctly. Doctors had significantly higher scores on (OR = 4.39, 95% CI 1.67–11.45, *p* = 0.003), as did older staff and those who had worked abroad OR = 3.06, 95% CI 1.60–5.85, *p* < 0.001). Most HCWs knew about methods of transmission (92%), hand hygiene practice (99%), HAIs prevention by complying with protocols (93%), and reducing cross-infection by using gloves, masks, and aprons (97%) Only 24% of HCWs had received training in IPC
Raab et al. [45], Guinea	Assesses the knowledge and practices of HCWs towards Ebola virus amongst in public healthcare facilities	Multi-centre	102 HCWs (31 technical assistants, 30 nurses, 15 physicians, 14 midwives, and 12 others)	Survey: questionnaire; [(Hoy critical appraisal checklist, LOW risk of bias)]	Overall knowledge on viral hemorrhagic fever was good among 99% of all interviewed HCWs Only 40.2% thought they would accept an approved vaccine for themselves and 37.3% would accept this for their parents for viral hemorrhagic fevers Significantly more HCWs in rural than urban healthcare facilities of the prefecture lacked IPC training (42.9% vs. 21.7%; *p* = 0.029)
Russell et al. [21], United States	Explore factors for compliance with IPC practices at 2 healthcare agencies	Multi-centre	359 nurses	Survey: questionnaire; [(Hoy critical appraisal checklist, LOW risk of bias)]	Nurses demonstrated correct knowledge (mean = 0.85, SD = 0.09), however, knowledge of IPC practices was not associated with compliance Majority of nurses reported having received IPC training in the previous year, with more than 39.3% reporting having received IPC training in the previous 6 months. However, less than 18.1% of nurses had formal IPC certification
Shi et al. [46], China	Assess knowledge of HCWs in 2 Chinese mental health centers during the COVID-19 outbreak	Multi-centre	311 HCWs (141 psychiatrists and 170 psychiatric nurses)	Survey: questionnaire; [(Hoy critical appraisal checklist, LOW risk of bias)]	Majority (79.10%) reported having extensive knowledge of COVID-19 (82.97% for physicians vs 75.88% for nurses, *p* = 0.125), and 78.78% expressed confidence in their ability to protect themselves and their patients (84.39% for physicians vs 74.12% for nurses, *p* = 0.027) About 64.63% of HCWs had finished a COVID-19 training program. Apart from the training program organized by their hospitals, various media (including the internet, television, and newspapers) were also major sources of knowledge. Moreover, significantly more physicians (38.30%) obtained their relevant knowledge from medical journals compared with nurses (7.06%)
Tavolacci et al. [47], France	Compare knowledge of hand hygiene between HCWs	Multi-centre	1811 HCWs (physicians, nurses, nursing assistants and others)	A questionnaire; [(Hoy critical appraisal checklist, LOW risk of bias)]	Physicians had better knowledge about hand hygiene than other HCWs. Knowledge of antiseptic efficacy of hand hygiene was 68.5% in senior physicians, 37.5% in consultants, and 52.9% in registrars and residents With regards to alcohol hand rub, sources of information were: colleagues (43.3%), IPC practitioner (33.3%), head nurses (27.6%), poster (14.4%), hospital epidemiologist (9.1%), articles in hospital's newspaper (5.7%), intranet (4%), IPC committee (2.6%)
Temesgen et al. [48], Ethiopia	Assess knowledge of TB IPC among HCWs in 4 healthcare facilities	Multi-centre	313 HCWs (59 physicians, 175 nurses, and 79 other healthcare professionals)	Survey: questionnaire; [(Hoy critical appraisal checklist, LOW risk of bias)]	Majority [74.4%, 95% CI (69.6, 79.3)] were found to have good knowledge (≥ 60% correct answers). Only 34.2% of the HCWs knew that respirators can provide protection from inhaling mycobacterium tuberculosis bacilli and only 46% correctly identified that use of a fan (ventilator) minimizes the risk of TB infection Only 18.8% of the HCWs were trained on TB IPC. Of these, 45% were trained in the past year while 55% were trained in the past two or more years Training was the strongest determinant of TB IPC knowledge, AOR 3.386 and 95% CI (1.377, 8.330)
Tenna et al. [49], Ethiopia	Evaluate HCW knowledge about hand hygiene and TB IPC measures at 2 university hospitals	Multi-centre	261 HCWs (133 physicians and 128 nurses)	Survey: cross-sectional questionnaire; [(Hoy critical appraisal checklist, LOW risk of bias)]	Hand hygiene knowledge was fair (60%). TB IPC knowledge was excellent (more than 90% correct) Only 56% of HCWs correctly believed that gloves do not provide complete protection against acquiring or transmitting infection (71% of physicians vs. 41% of nurses, *p* < 0.05). Only 59% knew that an alcohol-based hand sanitizer was as effective as soap and water when the hands were not visibly dirty (51% of physicians vs. 68% of nurses, *p* < 0.05) Only 50% of HCWs reported receiving hand hygiene training and only 30% thought their supervisors stressed the importance of hand hygiene
Trigg et al. [50], England	Evaluate HCWs knowledge regarding MRSA IPC precautions	Single centre	411 HCWs (47 physicians, 270 nurses, and 94 other health professionals)	Survey: cross-sectional questionnaire on the current guidelines for MRSA infection (Joint Working Party, 2006); [(Hoy critical appraisal checklist, LOW risk of bias)]	Staff showed high levels of knowledge on the IPC precautions required when caring for patients with MRSA (i.e., 84% HCWs knowledge was above 5), but some were confused about the level of isolation required for these patients (i.e., 35% of staff indicated isolation for some MRSA patients) Less than 46% of all HCWs had received any formal teaching. Highest percentage of HCWs who received education were doctors and unregistered nurses (51% and 53% respectively). Hotel services staff received the least, at 21%; 57% of HCWs felt that they had not received adequate education about MRSA with only doctors satisfied with the amount of education received

AOR, adjusted odds ratio; CDC, Centres for Disease Control and Prevention; CI: confidence intervals; COVID-19, coronavirus disease 2019; CVCs, central venous catheters; EDs, emergency departments; HAIs, health associated infections; HBV, hepatitis B virus; HCV, hepatitis C virus; HIV, human immunodeficiency virus; ICU, intensive care unit; IPC, infection prevention and control; MERS-CoV, Middle East Respiratory Syndrome Coronavirus; MRB, multi-resistant bacteria; MRSA, methicillin-resistant staphylococcus aureus; NSSIs, needle-stick and sharp injuries; OR: odds ratio; PEP, post-exposure prophylaxes; PPE, personal protective equipment; TB, tuberculosis; WHO, World Health Organization

**Table 2:** Summary of the characteristics of the included studies that have highlighted potential factors influencing compliance to the IPC precautions among HCWs (n = 16), 2006–2021

Author, year, study location	Study aim	Setting	Responded population	Methodology; and [assessment of study risk of bias (tool used; finding)]	Key findings
Abeje et al. [28], Ethiopia	Evaluate hepatitis B vaccination knowledge among HCWs	Multi-centre	374 HCWs (nurses, health officers, medical doctors, dentists, and laboratory technologists)	Survey: cross-sectional questionnaire; [(Williamson critical appraisal of qualitative evidence, LOW risk of bias)]	Hepatitis B vaccination status of HCWs was low
Albano et al. [16], Italy	Assess knowledge towards influenza A/H_1_N_1_ and the vaccination among HCWs	Multi-centre	600 HCWs (physicians, nurses and others)	Survey: cross-sectional questionnaire; [(Hoy critical appraisal checklist, LOW risk of bias)]	Only 16.7% have received the influenza A/H_1_N_1_ vaccination and HCWs with more fear of contracting influenza A/H_1_N_1_, those considering vaccine more useful and less dangerous were more likely to receive vaccine
Aloush et al. [19], Jordan	Assess compliance of HCWs with the with the CLABSIs IPC guidelines at 58 Middle Eastern hospitals on ICUs	Multi-centre	HCWs in 58 hospitals in the ICUs in three Middle Eastern countries (Jordan, Saudi Arabia and Egypt)	Observational; [(Hoy critical appraisal checklist, LOW risk of bias)]	Hospitals’ characteristics, lower number of beds and a lower patient-to-nurse ratio were related to higher compliance A significant lack of compliance was found in the item of continuing education. Only 14 hospitals had an active continuing education department that provided training and education for the staff on a regular basis
Alsahafi et al. [43], Saudi Arabia	Assess knowledge of HCWs to MERS-CoV	Multi-centre	1216 (687 nurses, 267 physicians, and 262 other HCWs)	Survey: questionnaire; [(Hoy critical appraisal checklist, LOW risk of bias)]	Compliance with immunization recommendations was poor (59.5% for annual influenza vaccine, 74.4% for meningococcal vaccine, and 50.4% for hepatitis B)
Amoran et al. [53], Nigeria	Assess compliance of HCWs with universal precautions in hospital environment	Single centre	421 HCWs (52 doctors, 78 nurses, 54 laboratory scientists, 53 pharmacists, 57 community health workers, 74 hospital orderlies, and 53 other professions)	Survey: cross-sectional questionnaire; [(Williamson critical appraisal of qualitative evidence, LOW risk of bias)]	Major reason for noncompliance to universal precautions is the nonavailability of the equipment. Higher compliance in HCWs who are exposed to blood products and body fluid (*p* = 0.03), public HCWs when compared to private HCWs (*p* = 0.001), among those working in secondary and tertiary facilities compared to primary healthcare centers (*p* = 0.001) and urban areas when compared to rural areas (*p* = 0.02) Knowledge of National policy on injection safety was not associated with practice of universal precaution among HCWs (*χ*^2^ = 0.404, *p* = 0.39); and recent training in IPC was not associated with the practice of universal precaution (*χ*^2^ = 0.013, *p* = 0.70)
Ashraf et al. [30], United States	Assess compliance with the 2002 CDC hand hygiene guidelines in nursing home settings	Multi-centre	1143 HCWs (386 nursing assistants, 375 nurses, and 382 other healthcare professionals)	Survey: questionnaire; [(Hoy critical appraisal checklist, MODERATE risk of bias)]	Lack of adherence to hand hygiene was due to absence of alcohol-based hand rub or absence of nearby sink or soap and paper towels (*p* < 0.001) Employees who reported receiving periodic education were significantly more likely to report washing hands when they are visibly dirty, when they are not visibly dirty, and after the use of gloves (*p* = 0.039, *p* = 0.002, and *p* < 0.001, respectively)
Assefa et al. [17], Ethiopia	Evaluate knowledge of HCWs about hand hygiene practices, utilization of PPE, and PEP, healthcare waste management practices, and instrument disinfection practice	Multi-centre	171 HCWs (about 83 were nurses)	Survey: questionnaire; [(Williamson critical appraisal of qualitative evidence, LOW risk of bias)]	The odds of safe practice were higher in participants who received IPC training (AOR: 2.4; 95% CI 1.01–4.75) but lower among HCWs who are working in the facility which has no continuous water supply (AOR = 0.48; 95% CI 0.21–0.83)
Chuc et al. [31], Vietnam	Assess and compare HCWs knowledge and self-reported practices of IPC in a rural and an urban hospital	Multi-centre	339 HCWs (nurses, midwives, physicians and cleaners)	Survey: cross-sectional questionnaire; [(Hoy critical appraisal checklist, LOW risk of bias)]	Self-reported practices in the urban hospital were likely to be better than in the rural one (*p* = 0.003). The two leading reasons for IPC noncompliance were emergencies (rural hospital: 75.7%, urban hospital: 75.9%) and high workload (rural hospital: 58.3%, urban hospital: 57.4%). Lack of equipment or soap was one of the most frequent reported reasons, followed by dry hands and allergies
Desta et al. [54], Ethiopia	Examine the knowledge and practice of HCWs on IPC and its associated factors among health professionals	Single centre	150 HCWs (21 Physician, 83 nurses, 18 midwives, 3 health officers, 13 lab technicians, 12 others)	Survey: cross-sectional questionnaire; [(Hoy critical appraisal checklist, LOW risk of bias)]	Majority of the HCWs (71.34%) doesn’t vaccinate for the common pathogen
Flores et al. [56], England	Evaluate the effect glove use has on HCWs' compliance with hand hygiene in 2 district general hospitals	Multi-centre	Doctors, nurses and healthcare assistants	Observational; [(Hoy critical appraisal checklist, LOW risk of bias)]	High rate of glove overuse (defined as the use of gloves when not required) (42%) might been a component of poor hand hygiene compliance
Ganczak et al. [26], Poland	Evaluate factors associated with the PPE use compliance and noncompliance among surgical nurses at 18 hospitals	Multi-centre	601 surgical nurses	Survey: questionnaire; [(Hoy critical appraisal checklist, LOW risk of bias)]	Compliance to PPE use was highest in the municipal hospitals and in the operating rooms (mean: 12.1 ± 4.7, *p* < 0.0001). Nurses who had fear of acquiring HIV were more likely to be compliant (mean: 12.0 ± 4.9, *p* < 0.005). Significantly higher compliance was found among nurses with previous training in IPC (mean: 12 ± 4.6, *p* < 0.009) or experience of caring for an HIV patient (mean: 12.9 ± 4.5, *p* < 0.0001). Most commonly stated reasons for noncompliance were non-availability of PPE (37%), conviction that the source patient was not infected (33%) and concern that following recommended practices actually interfered with providing good patient care (32%)
Geberemariyam et al. [18], Ethiopia	Assess knowledge of HCWs towards IPC	Multi-centre	648 HCWs (physicians, nurses, midwives, anesthetists, laboratory technicians, laboratory technologists, pharmacists, pharmacy technicians, and radiographers)	Survey: cross-sectional questionnaire; [(Hoy critical appraisal checklist, LOW risk of bias)]	There was a strong linear correlation between HCWs IPC knowledge score and the practice score (Pearson correlation coefficient = 0.703, *p* < 0.001). In addition, HCWs who have ever taken training on IPC were about 5.31 times more likely to practice safe infection prevention than those who have not received training (AOR = 5.31, 95% CI 2.42, 11.63)
Iliyasu et al. [8], Nigeria	Explore compliance of IPC among HCWs in a tertiary referral center	Single centre	200 HCWs (152 nurses and 48 doctors)	Survey: cross-sectional questionnaire; [(Hoy critical appraisal checklist, LOW risk of bias)]	About 52% of doctors and 76% of nurses (*p* = 0.002) always practice hand hygiene in between patient care. Knowledge on the risk of transmission of BBDs is related to higher compliance with PPE use (r = − 0.004, *p* < 0.001)
Loulergue et al. [51], France	Evaluate HCWs knowledge regarding occupational vaccinations (HBV, varicella and influenza)	Single centre	580 HCWs (physicians, nurses, nurses’ assistants)	Survey: cross-sectional questionnaire; [(Hoy critical appraisal checklist, LOW risk of bias)]	Influenza vaccination rate for 2006–2007 was 30% overall, ranging from 50% among physicians to 20% among paramedical staff (*p* < 0.05). Physicians based their refusal on doubts about vaccine efficacy, although paramedics feared side effects
Michel-Kabamba [42], Democratic Republic of the Congo	HCWs knowledge on COVID-19-related clinical manifestations and patient care approach was assessed using WHO’s “Exposure Risk Assessment in the Context of COVID-19” questionnaire	Multi-centre	613 HCWs (27.2% were medical doctors and 72.8% were other categories of HCWs)	Survey: cross-sectional questionnaire; [(Hoy critical appraisal checklist, MODERATE risk of bias)]	Practices scores were relatively low. About 55% of HCWs complied with good practices; 49.4% wore masks consistently and, surprisingly, only 54.9% used PPE during contact with patients HCWs from towns already affected by the COVID-19 epidemic being more likely to comply with good practices (AOR, 2.79; 95% CI 1.93–4.06) Only 27.7% of HCWs were willing to receive a COVID-19 vaccine when it is available
Ogoina et al. [34], Nigeria	Examine compliance of HCWs with standard precautions in two tertiary hospitals	Multi-centre	290 HCWs (111 doctors, 147 nurses and 32 laboratory scientists)	Survey: cross-sectional questionnaire; [(Hoy critical appraisal checklist, LOW risk of bias)]	Compliance of laboratory scientists (46.2%), house officers (49.2%), and staff nurses (49.2%) were lower than those of consultants (53%), resident doctors (56.9%) and principal nursing officers (50.7%); *p* < 0.0001) Lack of enough facilities and resources to practice IPC (66.1%), absence of training on IPC (52.4%), lack of IPC committee (38.9%) and excess workload (34.8%) were main challenges to prevent HCWs from practice of standard precautions
Parmeggiani et al. [3], Italy	Assess HCWs compliance with IPC in the EDs	Multi-centre	307 HCWs (nurses, physicians and other healthcare professionals)	Survey: cross-sectional questionnaire; [(Hoy critical appraisal checklist, LOW risk of bias)]	Two independent predictors of compliance were positively associated: fewer patients cared in a day (OR = 0.97; 95% CI 0.95–0.99) and know that hands hygiene measures after removing gloves is a control measure (OR = 8.09; 95% CI 2.83–23.1)
Russell et al. [21], United States	Explore factors for compliance with IPC practices at 2 healthcare agencies	Multi-centre	359 nurses	Survey: questionnaire; [(Hoy critical appraisal checklist, LOW risk of bias)]	A high level of IPC compliance (mean = 0.89, [SD] = 0.16). Positive association of attitude with level of compliance (*p* = 0.001) Older nurses, non-Hispanic black nurses, and nurses with IPC certification reported greater compliance with IPC practices than younger nurses (β = 0.003, *p* < 0.05), non-Hispanic white nurses (β = 0.072, *p* < 0.001), and nurses without IPC certification (β = 0.047, *p* < 0.05)
Shah et al. [55], England	Identify behaviors of HCWs that facilitated noncompliance with IPC practices at 3 tertiary hospitals	Multi-centre	Doctors, pharmacists, nurses and midwives	Semi-structured interviews; [(Williamson critical appraisal of qualitative evidence, LOW risk of bias)]	Attribution of responsibility, prioritization and risk appraisal, and hierarchy of influence depict HCWs’ different motivations for compliance with IPC practice
Tavolacci et al. [47], France	Compare compliance with hand hygiene between HCWs	Multi-centre	1811 HCWs (physicians, nurses, nursing assistants and others)	A questionnaire; [(Hoy critical appraisal checklist, LOW risk of bias)]	Use of hand hygiene differed according to professional category and experience. Knowledge of hand hygiene efficacy (88.5% by physicians vs 83.8% by other HCWs, *p* = 0.001), opinion that hand hygiene is easy to use (97.3% by physicians vs 94.9% by other HCWs, *p* = 0.37) and hand hygiene has acceptable skin tolerance (68.8% by physicians vs 54.3% by other HCWs, *p* = 0.004) improved hand hygiene compliance
Temesgen et al. [48], Ethiopia	Assess knowledge of TB IPC among HCWs in 4 healthcare facilities	Multi-centre	313 HCWs (59 physicians, 175 nurses, and 79 other healthcare professionals)	Survey: questionnaire; [(Hoy critical appraisal checklist, LOW risk of bias)]	Knowledge about TB IPC was the strong predictor of good TBIC practice, AOR 10.667 and 95% CI (5.769–19.721)
Tenna et al. [49], Ethiopia	Evaluate HCW compliance with hand hygiene and TB IPC measures at 2 university hospitals	Multi-centre	261 HCWs (133 physicians and 128 nurses)	Survey: cross-sectional questionnaire; [(Hoy critical appraisal checklist, LOW risk of bias)]	Self-reported TB IPC practice was suboptimal Physicians reported performing hand hygiene 7% and 48% before and after patient contact, respectively Barriers for performing hand hygiene included lack of hand hygiene agents (77%), sinks (30%), proper training (50%), and irritation and dryness (67%) caused by hand sanitizer

AOR, adjusted odds ratio; BBDs, blood borne diseases; CDC, Centres for Disease Control and Prevention; CI: confidence intervals; CLABSIs, central Line associated bloodstream infections; COVID-19, coronavirus disease 2019; EDs: emergency departments; HBV, hepatitis B virus; HIV, human immunodeficiency virus; ICU, intensive care unit; IPC, infection prevention and control; MERS-CoV, Middle East Respiratory Syndrome Coronavirus; OR: odds ratio; PEP, post-exposure prophylaxes; PPE, personal protective equipment; SD: standard deviation; TB, tuberculosis; WHO, World Health Organization

### Data analysis

Preliminary screening of eligible studies revealed large considerable heterogeneity in terms of participants, setting, sample size, response rates and outcome measures. Primary analysis of studies was, therefore, limited to qualitative synthesis, allowing a detailed analysis of the data to be performed.

## Results

**Fig. 2:**
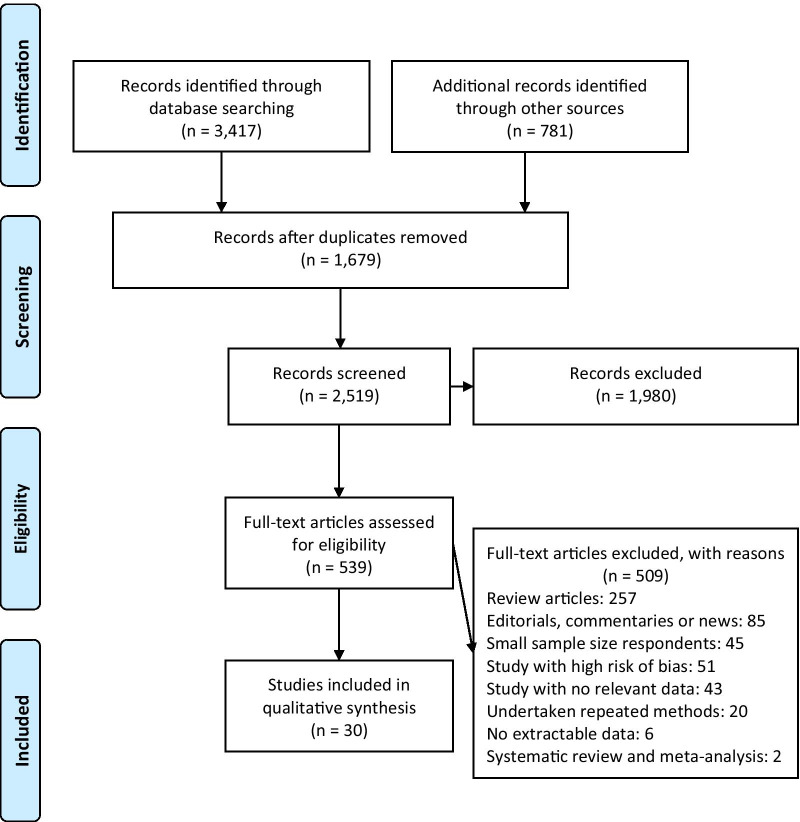
Flow diagram of studies included in the systematic review

A total of 3417 publications were retrieved; of which 30 were eligible for final analysis. The PRISMA chart for the studies included is displayed in Fig. 2. Research methodologies were predominantly self-report questionnaires (n = 26), some of which were cross-sectional surveys using a questionnaire (n = 14) and observation techniques (n = 2). Other methodologies were semi-structured interview studies (n = 5) targeted at individual and institutional levels. The sampling method in most studies was convenience sampling (n = 22). Thirty studies conducted on 16,081 HCWs entered the final stage. The majority of the studies were conducted in high-income countries [n = 16; (United States = 4, England = 3, Italy = 2, France = 2, Saudi Arabia = 1, Poland = 1, China = 1, and European countries = 1)]; while others originated from lower-middle-income countries [n = 6; (Nigeria = 3, Vietnam = 1, India = 1, and Nepal = 1)], and low-income countries [n = 8; (Ethiopia = 6, Guinea = 1, and Democratic Republic of Congo = 1)]. Samples ranged from 102 HCWs to multiple sites. Most of the sampled studies were from hospital settings (28 studies), at least 9 of which focused on acute hospital settings. The majority of studies were undertaken in intensive or critical care units, outpatient departments, emergency departments, primary health care centres, maternity units or paediatric or neonatal hospitals, with others taking place in long-term care facilities, medicine and surgery, cardiac, renal, dental, urology, and psychiatry.

All studies had a high level of quality (low bias), and except for 3 studies [30, 41, 42], all others used nonstandardized questionnaire survey instruments. Most of the studies included nurses (29 studies) or doctors (23 studies). Other types of HCWs included in the studies were allied healthcare workers such as pharmacists and pharmacy technicians, dentists, midwives, laboratory specialists and technicians, laboratory technologists, radiographers, community health workers, and health officers; ancillary staff with responsibility for patient care, such as porters and domestic workers. The sampled studies focused on HCWs’ knowledge and compliance of standard or universal precautions [13], awareness of IPC national and international guidelines or recommendations [5], NSSIs precautions [2], and urinary catheters- and CVCs-related infections [2], healthcare waste handling [1]; and views and experiences with regards to IPC for TB (3 studies), influenza [3], COVID-19 [2], HBV [2], H_1_N_1_ [1], MERS [1] MRSA [1], Ebola [1] or varicella [1]. Summary of the characteristics of the included studies that have assessed the knowledge of IPC among HCWs (n = 26) and highlighted potential factors influencing compliance to the IPC precautions (n = 22) is available in Tables 1 and 2, respectively. Additional findings of descriptive and professional guidelines are noted throughout the text.

### Knowledge of IPC among HCWs

Twenty-six out of 30 of the included studies examined knowledge level. In most studies, the HCWs IPC knowledge was measured by question items in which responses were answered in a point Likert scale or yes and no options. The level of knowledge in different studies had been mainly categorized as aware, adequate, high, knowledgeable, good and fair. We mentioned the most common level of knowledge in each study. Out of the 26 studies, level of knowledge was characterised as: “aware” (range: 77.9–94.7%, n = 5 studies), “adequate” (range: 70.8–84.7%, n = 2 studies), “high” (range: 80.2–84%, n = 3 studies), and “knowledgeable” (range: 53.7–76%, n = 2 studies); respectively. Four studies also reported level of knowledge as excellent (≥ 90%, n = 1), good (74.4–99%, n = 2), and fair (60%, n = 1). In the group of 3 studies, the most common level of knowledge was characterized as low (range: 25–34%) or poor (50%) (Table 1).

Findings regarding knowledge of IPC measures were mixed. On the one hand, the level of HCWs knowledge on IPC was adequate, good, excellent and/or high in relation to standard precautions (use of gloves, mask, gowns and protective eye wear), hand hygiene, IPC measures for TB, MRSA, MERS-CoV, COVID-19 and Ebola, and care pertaining to urinary catheters [8, 27, 29, 34, 43–50]. On the other hand, although awareness of children's hospital HCWs toward mandatory influenza vaccination was high, knowledge of HCWs about the occupational vaccinations, namely, HBV, varicella and influenza was low [51–53].

Analyses of the reviewed studies indicated specific variation in the knowledge of IPC from one country to another. For instance, in comparison with the high mean knowledge of HCWs in Italy and Nepal on main methods of transmission for microorganisms, only half of the HCWs in an Indian study understood disease transmission through NSSIs and less than one-third of Italian HCWs knew that they could transmit these infections to a patient [3, 29, 44]. Low level of knowledge on modes of transmission among HCWs was also evident in another Italian and a Nigerian study [8, 16]. In a 22-European country survey, the mean score of the nurses' knowledge on the CDC guidelines for preventing CVCs-related infections was low [41]. A British study demonstrated that HCWs never had the correct understanding about the level of isolation required for MRSA patients [27]; and an American study shown nurses and their aides were less aware of research-proven recommendations for urinary catheter patients [50]. Two Ethiopian studies found low percentage of HCWs were knowledgeable towards IPC [19]; and only few HCWs knew that respirators can provide protection from inhaling TB pathogen and the use of ventilator can minizine risk of TB infection [48]. Furthermore, two Nigerian studies shown HCWs had poor awareness of the risks of infection following exposure to BBDs and injection safety; and HCWs were unable to recognize vaccination, post exposure prophylaxis, surveillance for emerging diseases, and the national injection safety policy and policy on sharps disposal [8, 34].

Sources of information for HCWs on knowledge of IPC included public media [16, 42, 46]; healthcare providers (IPC practitioners, hospital epidemiologists and nurses) [16, 46, 47]; internet and intranet [16, 42, 46, 47]; medical journals [3, 43, 46]; Ministry of Health’s websites, memos and helplines [42, 43]; hospital’s newspapers [46, 47]; IPC committee [47]; posters [47]; educational courses [3]; and hospital training [46]. HCWs reported learning from didactic formal [in-services, lectures, and nursing school and nurse aides' courses] and informal [prior experience, nurse supervisors, co-workers, and facility policies] methods; and some HCWs gained their knowledge both informally and formally [27].

Several associations were found between HCWs' knowledge and other variables (such as experience, training, working abroad, availability of IPC guidelines, participation in an IPC committee, and receiving information through scientific journals) [3, 16–18, 34, 41, 48, 54]. HCWs who taking IPC training and education [17, 18, 34, 48, 54], having long work experience [17, 18, 41, 54], had IPC guidelines [17, 18], received information through medical journals [3, 16] and participated in an IPC committee [18] were more likely to be knowledgeable on IPC. HCWs with an educational level of bachelor, master or above had more knowledge on IPC than HCWs holding diplomas [54]. Being a doctor or a nurse, rather than a nurse assistant, midwife, laboratory technologist or scientist, pharmacist, community health worker, house officer, and other type of HCWs was consistently associated with more knowledge of injection safety, infection preventive measures, CDC hand hygiene guidelines, and influenza vaccine recommendations [3, 8, 30, 31, 34, 42, 47, 51].

### Factors influencing compliance of HCWs with IPC

Most studies assessed compliance with IPC by means of self-reporting, using self-developed questionnaires and few studies measured compliance using direct observation by a trained observer (Table 2). Several factors that may affect HCWs' compliance and noncompliance with the IPC measures were reported in many research studies [3, 8, 17, 19, 21, 26, 30, 34, 47, 49, 53, 55, 56]. Three of the major factors prompting HCWs to comply with the IPC measures were knowledge, education and training, and experience [8, 17–19, 26, 30, 34, 47, 49]. For instance, more awareness of the IPC benefits and procedures and the perception of risk associated with not following IPC recommendations motivated HCWs to be more compliant [3, 8, 47]. HCWs who reported receiving enough training and education on IPC were much more compliant [17–19, 26, 34, 49]. Also, HCWs who cared longer for patients with a history of infective diseases or participated in IPC committees were more adherent to IPC practices [26, 34, 42, 47]. Being a doctor rather than a nurse was associated with lower compliance with hand hygiene guidelines, PPE use and IPC practices [21, 26]. Compliance was the lowest in ICUs compared with non-ICU wards or surgical wards [17, 26], and higher when HCWs were working at public, secondary and tertiary healthcare facilities [26, 53] and during performing procedures that carried more exposure to blood products and body fluid or when HCWs were fearful of acquiring BBDs [26, 53]. Compliance of HCWs with IPC in the urban hospitals was better than in the rural ones [31, 53]. Other factors identified for good adherence to IPC practices were older age, positive attitude and non-Hispanic black race [21].

Predictors of HCWs noncompliance included high workload [31, 34] and time constraints [34], more beds and/or higher patient-to-nurse ratio [3, 19], and professional category-specific [47]. Glove overuse seemed to reduce hand hygiene compliance [56]. Noncompliance of HCWs to occupational vaccinations recommendations was due to lack of fear of contracting the infection (e.g. influenza A/H_1_N_1_) [16], doubts about vaccine efficacy [51], belief that vaccine is useless or dangerous [16], and fear of vaccine side effects [51]. Reported barriers for HCWs to adhere with standard precautions included nonavailability of equipment (alcohol hand rub, nearby sink, soap or paper towels) [17, 26, 30, 31, 49, 53] and intolerable or difficult to use hand hygiene agents [47, 49]. Lack of implemented IPC protocols [16, 17, 49]; HCWs belief that patients pose no health risk on them or patients cannot be a source of infection as if they were asymptomatic or unaware that they are infected; or following IPC recommendations interferes with providing good patient care resulted in less IPC adherence [26].

## Discussion

There appears to be gaps in some HCWs' knowledge of occupational vaccinations (HBV, varicella and influenza), modes of transmission of infectious diseases (HBV, HCV, HIV, and influenza A/H_1_N_1_), the risk of infection from NSSIs, the understanding that needle and sharp safe practices are enough to protect against BBPs, and the CDC guidelines for preventing CVCs-related infections. Lack of knowledge of IPC among HCWs has been linked to the worsening of the healthcare delivery outcomes [1, 7, 57]. For instance, insufficient knowledge of HCWs about occupational vaccinations resulted in low coverage of HCWs for hepatitis B [28, 43], influenza A/H_1_N_1_ [16, 43, 51], meningococcal [43], and COVID-19 [42] vaccines. Majority of HCWs does not vaccinate against the common pathogens [54]. These unsafe practices may increase exposures and infections among HCWs and impede control of infectious disease outbreaks [58]. Therefore, as vaccine coverage is associated with knowledge [51], education and training should be strengthened to increase the adhesion of HCWs to vaccinations. Many studies shown HCWs received insufficient training and education on IPC and they needed more education and training [34, 42–45, 48–50, 53]; and in some studies, HCWs admitted that, away from their professional education, they did not receive any training or orientation on IPC in the prior year or were not sure whether they had received training [17, 30, 59, 60]. Some HCWs stated that training was only available for administrators, not frontline HCWs [61]. Education and training is recommended as a core component for effective IPC programmes by the World Health Organization [WHO] [62]. Effective education and training of HCWs on IPC is beneficial and has reduced HAIs and combated antimicrobial resistance (AMR) considerably [23, 24, 63, 64]. Educational programmes have been reported as an essential ingredient for success in IPC strategies, including increasing HCWs acceptance of occupational vaccinations [51], the control of ventilator-associated pneumonia [65, 66], reducing needlestick injuries [67], and the implementation of isolation precautions [68]. There are also reports on the effective use of education for hand hygiene promotion strategies outside the acute hospital care setting [69]. It is important, therefore, to continue to use the formal education programme as one feature of the implementation strategy for IPC improvement in health care. There was a positive correlation between good knowledge and compliance among the HCWs [8, 18, 70–73]. Training and education are necessary if full compliance to IPC guidelines is to be achieved [18, 30, 74]. Therefore, health care facilities need to arrange training sessions for all HCWs to improve their knowledge and to improve their level of compliance.

Knowledge of IPC among HCWs other than physicians and nurses was lower in comparison with physicians and nurses, and their role in tackling HAIs is pivotal. Basically, this could be due to lower level of academic education and training about IPC in HCWs other than physicians or nurses. The role of HCWs other than physicians and nurses in hospital IPC is usually underestimated [75, 76] although they themselves and their work can be a vector of infection transmission in hospitals. HCWs other than physicians and nurses may be in close contact between patients; physicians, and/or nurses; high concentrations of medically-vulnerable populations, combined with physical movement between treatment areas; which may facilitate HAI spread within health care institutions and the community. Therefore, WHO guidelines recommend that IPC education and training should be in place for all HCWs using team- and task-based strategies, including bedside and simulation training [62]. Inclusion of educational curricula and continuing refresher education programs about IPC can be recommended for HCWs other than physicians and nurses to ensure a thorough knowledge and understanding of IPC. Although educational initiatives have so far not been consistently associated with good IPC practices [61, 77–79], targeted materials and training can help ensure understanding among HCWs and healthcare facility visitors [80]. Since different categories of HCWs may have different information needs, it is recommended that IPC training sessions be tailored to the specific target audience, e.g., medical staff versus cleaning services staff. Education is important to address HCWs’ concerns, fears, stigmas and incorrect assumptions regarding transmission or prevention of HAIs.

While HAIs burden is already demanding in developed countries, magnitude of the problem is intensified in healthcare organizations where basic IPC measures are not available mainly due to limited financial resources. Familiarity with IPC measures is challenging even in highly resourced countries and may appear an unrealistic goal in everyday care in resource constrained countries with financial constraints [5]. Limited resources are a common contributor to poor IPC practices [30, 61, 81]. Therefore, simple and affordable preventive measures such as hand hygiene should be adapted in the healthcare settings of resource constrained countries. Hand hygiene is the initial step towards successful IPC and still remains the basic and most effective measure to prevent pathogen transmission and infection. Simple hand hygiene when performed well can reduce the prevalence of HAIs substantially [5].

HCWs has been known to get infected during disease outbreaks and pandemics such as MERS-CoV outbreak [82] as well as the COVID-19 pandemic [83] due to poor compliance with the basic IPC measures. IPC recommendations in response to Severe Acute Respiratory Syndrome and other corona viruses should be informed by these previous well-established IPC knowledge and experiences, and perhaps infection preventive guidelines influenced by them to a certain extent; and should include standard precautions and droplet or airborne precautions [62].

Organized national programs or campaigns shown to highly promote IPC and ensure effective implementation of strategies and guidelines and have a favourable effect on HCWs' IPC knowledge and improved compliance [64, 79, 84]. National interventions for IPC undertaken in the context of a high-profile political drive can reduce selected HAIs [84].

Unfortunately, good knowledge does not necessarily predict good practice [8, 33, 34, 53].

More confounding variables of good IPC practice other than knowledge or experience exist. Nonavailability of resources, high workload and time limitation have been reported as the main factors influencing HCWs' compliance with IPC practice [26, 30, 34, 49, 53]. Factors that impact on compliance is organized into three overarching domains: organisational, environmental and individual factors [85]; which allows HCWs, managers and policy makers to see clearly where strategies need to be implemented to facilitate compliance and support HCWs. Reported compliance with IPC in the public, secondary and tertiary hospitals was likely to be better than in the private or primary ones. This might be possibly related to poorer conditions for IPC at the private or primary hospitals. Poor compliance with IPC practices has been observed both in high- and low-income settings and across health settings [22, 55]. Identifying roles and responsibilities of a team and making IPC initiatives its top priority in addition to influence by peer pressure group could play a role in determining HCW's different motivations for compliance [55].

HCWs seem to be selective in adhering to IPC measures rather than practicing comprehensive safe standard precautions when engaging in contact with patients which may result in an unnecessary risk. In particular, during performing procedures that carry more exposure to blood products and body fluid or when dealing with sharps, compliance is good. While many researchers have investigated in the area of compliance with IPC guidelines and reasons for non-adherence [22, 86], with a lack of knowledge regularly being identified by staff as affecting their compliance, the opinions of HCWs about what would improve their own practice may need to be questioned further.

IPC behaviour varies significantly among HCWs, thus suggesting that individual features could play a role in determining behaviour [20, 26, 27]. There is a danger in ignoring all-important ‘individual differences’ and a call to limit this approach within health psychology has previously been made [87]. To improve HCWs’ compliance with practices, IPC should learn from the behavioural sciences [88]. Social psychology attempts to understand these features, and individual factors such as social cognitive determinants may provide additional insight into IPC behaviour [89]. Application of social cognitive models and psychological principles in intervention strategies has resulted in a change towards positive behaviour in IPC [90–92]. Current models that help to explain human behaviour can be classified on the basis of being directed at the intrapersonal, interpersonal, or community levels [32, 89]. Intrapersonal factors are individual characteristics that influence behaviour such as knowledge, attitudes, beliefs and personality traits. Interpersonal factors include interpersonal processes and primary groups, i.e., family, friends and peers, who provide social identity, support and role definition. Community factors are social networks and norms that exist either formally or informally between individuals, groups and organizations [32, 89].

Multifaceted approach (e.g. education, training, observation, feedback, easy access to hand hygiene supplies, dedication of financial resources, praises by superior, strong hospital leadership, prioritization to IPC needs, collaborating with a private advertising firm in a marketing campaign and active participation at institutional level) is highly suggested to reduce HAIs by improving compliance among HCWs with IPC measures [93, 94].

## Limitations

Subject variability and study outcomes meant to be measured to affect the validity of this review and invalidate the findings. Lack of homogeneity among the studies included. We were not able to perform any type of meta-analysis because of the large methodological differences. There are methodologic issues with almost all the included studies in relation to credibility, transferability, and confirmability. The research methodologies were predominantly cross-sectional surveys using a questionnaire; some of which were self-report questionnaires and observation techniques. Clearly, this does raise some methodological issues in terms of the reliability of observational data and self-report questionnaires, and the probability of observer and social desirability affecting the results. The majority of the participants included in these research studies were nurses or doctors. The exclusion of studies published in languages other than English may have impacted on the richness of the data included in this review. Furthermore, some of the studies failed to report on the type of instrument used for obtaining the data or on how observers were trained. This makes comparison and interpretation of the results difficult, not only in this review but in general for researchers interested in knowledge of IPC amongst HCWs and factors influencing compliance studies.

## Conclusion

This review intended to discuss literature on knowledge of IPC among HCWs and factors influencing compliance. Overall, the level of HCWs knowledge on IPC seems to be adequate towards standard precautions, hand hygiene, and IPC measures for TB, MRSA, MERS-CoV, and COVID-19, and care pertaining to urinary catheters. There appears to be gaps in some HCWs' knowledge of occupational vaccinations (HBV, varicella and influenza), modes of transmission of infectious diseases (HBV, HCV, HIV, and influenza A/H_1_N_1_), the risk of infection from NSSIs, the understanding that needle and sharp safe practices are enough to protect against BBPs, and the CDC guidelines for preventing CVCs-related infections. Several factors may affect HCWs' compliance and noncompliance with the IPC measures: knowledge, education and training, experience, lack of supplies (alcohol hand rub, nearby sink, soap or paper towels), working in ICU or surgical ward, working at public or secondary or tertiary hospital, and working for a patient with exposure to blood or body fluid. Barriers to comply with IPC may include workload, insufficient time, professional category and low patient-to-nurse ratio. It is highly suggested that adopting a multifaceted approach to IPC improvement intervention strategies has been shown to reduce HAIs and improve compliance among HCWs with IPC measures.

## Data Availability

Data are available upon request, please contact author for data requests.

## Abbreviations

IPC: Infection prevention and control
HCWs: Healthcare workers
HAIs: Healthcare-associated infections
PRISMA: Preferred Reporting Items for systematic reviews and meta-Analyses
PPE: Personal protective equipment
WHO: World Health Organization
CDC: Centres for Disease Control and Prevention
ICUs: Intensive care units
BBPs: Bloodborne pathogens
NSSIs: Needle stick and sharps injuries
HBV: Hepatitis B virus
HCV: Hepatitis C virus
HIV: Human immunodeficiency virus
CVCs: Central venous catheters
BBDs: Blood borne diseases
